# Accuracy of LLMs in medical education: evidence from a concordance test with medical teacher

**DOI:** 10.1186/s12909-025-07009-w

**Published:** 2025-03-26

**Authors:** Vinaytosh Mishra, Yotam Lurie, Shlomo Mark

**Affiliations:** 1 Datta Meghe Institute of Higher Education & Research, Nagpur, Maharashtra India; 2 Gulf Medical University, Ajman, UAE; 3 Ben-Gurion University of the Negev, Be’er Sheva, Israel; 4 Shamoon College of Engineering, Ashdod, Israel

**Keywords:** Generative AI, LLM, Machine learning, Medical education

## Abstract

**Background:**

There is an unprecedented increase in the use of Generative AI in medical education. There is a need to assess these models’ accuracy to ensure patient safety. This study assesses the accuracy of ChatGPT, Gemini, and Copilot in answering multiple-choice questions (MCQs) compared to a qualified medical teacher.

**Methods:**

This study randomly selected 40 Multiple Choice Questions (MCQs) from past United States Medical Licensing Examination (USMLE) and asked for answers to three LLMs: ChatGPT, Gemini, and Copilot. The results of an LLM are then compared with those of a qualified medical teacher and with responses from other LLMs. The Fleiss’ Kappa Test was used to determine the concordance between four responders (3 LLMs + 1 Medical Teacher). In case of poor agreement between responders, Cohen’s Kappa test was performed to assess the agreement between responders.

**Results:**

ChatGPT demonstrated the highest accuracy (70%, Cohen’s Kappa = 0.84), followed by Copilot (60%, Cohen’s Kappa = 0.69), while Gemini showed the lowest accuracy (50%, Cohen’s Kappa = 0.53). The Fleiss’ Kappa value of -0.056 indicated significant disagreement among all four responders.

**Conclusion:**

The study provides an approach for assessing the accuracy of different LLMs. The study concludes that ChatGPT is far superior (70%) to other LLMs when asked medical questions across different specialties, while contrary to expectations, Gemini (50%) performed poorly. When compared with medical teachers, the low accuracy of LLMs suggests that general-purpose LLMs should be used with caution in medical education.

**Supplementary Information:**

The online version contains supplementary material available at 10.1186/s12909-025-07009-w.

## Practice points

This study evaluates Large Language Models (LLMs) compared to experienced medical teachers.

The analysis examines the performance of three prominent LLMs—ChatGPT, Gemini, and Copilot.

The study employs Fleiss’ Kappa Test to statistically analyze the concordance between LLMs and human responses.

In discordance, Cohen’s Kappa test was used to find agreement between three Gen AI tools and a Medical Teacher.

Results reveal a significant difference in the performance between LLMs and medical teachers, highlighting potential limitations in using AI alone for medical education.

**Supplementary Information:**

The online version contains supplementary material available at 10.1186/s12909-025-07009-w.

## Introduction

The use of computers in medical education has significantly increased in the last few decades. Integration of technologies such as virtual reality (VR), augmented reality (AR), and computer-assisted learning (CAL) has been instrumental in imparting various aspects of training [1]. Integrating these technologies has transformed traditional pedagogical methods, enhancing the learning experience for medical students and professionals alike. This amalgamation facilitates the acquisition of complex skills and addresses the limitations of conventional training methods [2]. The e-learning tools, including three-dimensional resources, offer advantages over traditional training by enabling greater accessibility and flexibility [3]. By utilizing virtual space, students may effectively comprehend complex anatomical structures that are typically difficult to understand through textbooks or static representations. Computer-assisted learning programs have been successfully utilized in different medical fields, including dentistry, alongside surgical training. Karemore et al. stated that CAL could replicate genuine patient encounters, allowing dental students to get useful experience without any potential risks [4].

Moreover, it highlighted the significance of incorporating digital technology into dentistry education. It was observed that students prefer computer-assisted learning tools if they give concrete educational advantages [5]. Recent advancements in generative artificial intelligence (AI) have opened new opportunities in medical education.

### Generative AI in medical education

Generative artificial intelligence refers to the ability of artificial intelligence systems to produce text, photos, videos, or other types of data using generative models. This is typically done in response to specific prompts or inputs. Generative AI models acquire knowledge of the patterns and organization of their input training data, enabling them to produce novel data with comparable attributes [6]. Generative AI has been significantly influenced by methods like Generative Adversarial Networks (GANs) and extensive language models like BERT- Bidirectional Encoder Representations from Transformers and GPT- Generative Pre-train Transformers. These techniques have facilitated the practical application of generative AI in various fields, including medical education [7].

Open AI developed GPT, while BERT was used by Google AI earlier. Both fall under a wider umbrella of natural language processing (NLP) models known as Large Language Models (LLMs). LLMs are machine learning models that can understand and generate human language text and are called language models [8]. BERT aims mainly to comprehend text by considering the context before and after the target word. It is extensively utilized for jobs requiring profound text comprehension, such as classification and question answering. GPT is specifically engineered to generate text by accurately predicting the subsequent word in each sequence. It is mostly utilized for text generation jobs, like creating dialogues or summarizing content. Thus, GPT is expected to outperform others in generating coherent & creative text; hence, it is a powerful tool for content creation.

On the other hand, BERT, with its deep understanding of context, excels in tasks requiring nuanced comprehension and accurate information retrieval [7]. The architecture is compared to give the background when comparing two popular general-purpose LLMs, ChatGPT4 and Gemini. However, these models have significantly evolved and are expected to perform better in helping in medical education tasks such as providing answers to multiple-choice questions (MCQs). The differences between GPT and BERT are summarized in Table 1.

**Table 1:** Comparison of BERT and GPT

Feature	BERT	GPT
Directionality	Bidirectional	Unidirectional (left-to-right)
Architecture Focus	Encoder-only Transformer	Decoder-only Transformer
Primary Objective	Understanding context and relations in text	Generating coherent and contextually relevant text
Pre-training Tasks	Masked Language Model, Next Sentence Prediction	Language Modeling (predicting the next word)
Application Focus	Understanding and interpreting text	Generating text
Fine-tuning	Requires fine-tuning for specific tasks	It can be used with few-shot learning for various tasks
Use Cases	Text classification, question answering	Text generation, Summarization, Dialogue systems

### Specific LLMs and their current use

**Fig. 1:**
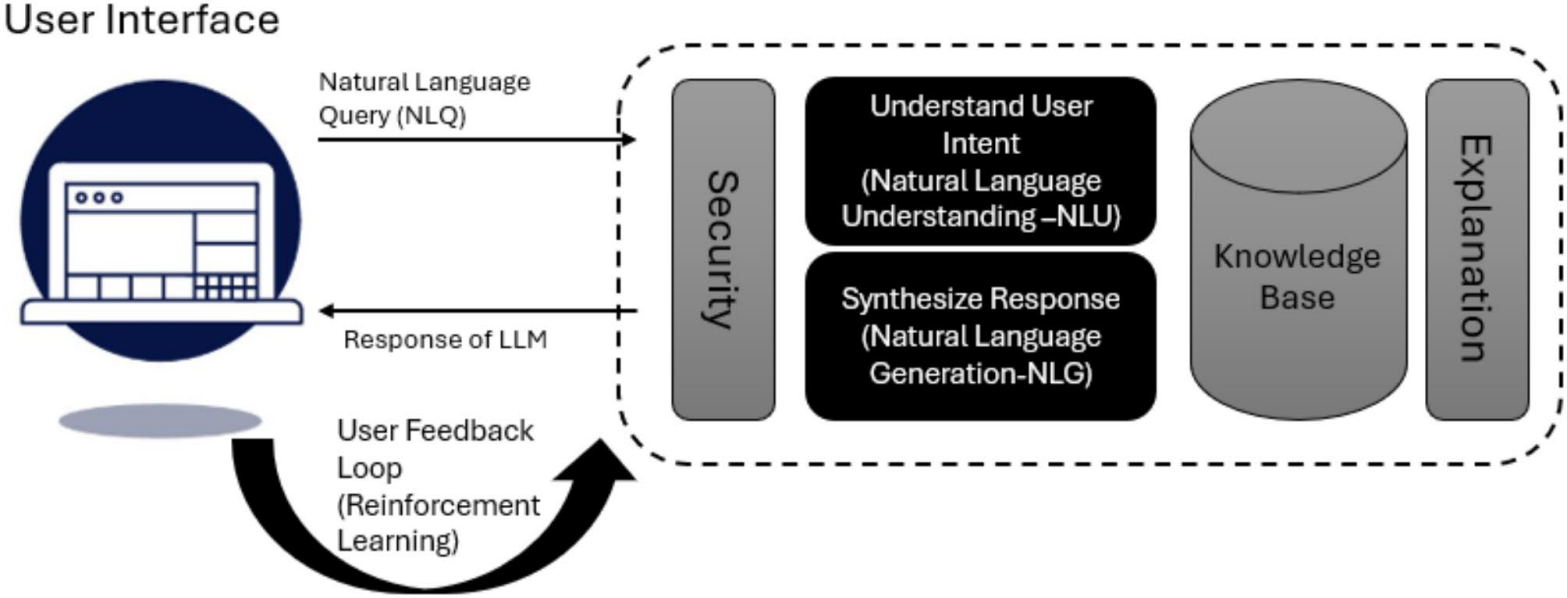
High-level View of LLM Architecture

Gemini can produce written content by using input provided by the user. The text can also be operated through a chatbot interface that follows a question-and-answer format. Translation of the text [9]. The Gemini models possess extensive multilingual capabilities, facilitating the translation and comprehension of over 100 languages. According to OpenAI, ChatGPT is trained to interact conversationally. Its dialogue format makes it possible to answer follow-up questions, admit mistakes, challenge incorrect premises, and reject inappropriate requests. GPT-4 uses code more sophisticatedly and is better at hard verbal tasks, while Gemini is better at explanations and searches. Gemini may be the optimal choice if you require assistance solving complex problems and comprehending profound concepts. However, if you require aid with routine activities or engaging in discussions, GPT-4 may be better suited to fulfil your requirements. The high-level chatbot architecture for LLMs, such as ChatGPT, is given in Fig. 1.

### Gap in literature

Recent literature highlights the transformative potential of large language models (LLMs) in medical education. Ishaaq and Sohail emphasize the role of AI chatbots in enhancing clinical decision-making, particularly in the context of reduced clinical exposure during the pandemic [10]. This aligns with findings from Al-Qerem et al., who explore health professions students’ attitudes towards AI, indicating a growing recognition of its educational benefits [11]. Furthermore, Wong et al. discuss ethical considerations and the implications of integrating tools like ChatGPT into medical curricula, suggesting that these technologies can augment traditional learning methods [12]. Overall, the integration of LLMs in medical education is seen as a necessary evolution to prepare future healthcare professionals effectively. Extant literature compares Gemini and ChatGPT’s utility in ophthalmology [13, 14]. A study comparing both LLMs in hypertension education found that ChatGPT outperformed Gemini in answering questions [15]. Another study compares a comparative analysis of ChatGPT, Gemini, and emergency medicine specialists in ESI triage assessment and again found that GPT-4 was found to have the highest correct triage rate. In contrast, Gemini had the highest over-triage rate. The highest under-triage rate was observed in emergency medicine specialists [16]. The study concluded that GPT-4 and Gemini can accurately triage critical and urgent patients rapidly. They further concluded that GPT-4 has been more successful in ESI triage for all patients. A scientific report published in Nature observes that Copilot is more accurate at interpreting biochemical data than Gemini and ChatGPT 3.5 [17]. ChatGPT-4 and CoPilot utilize GPT-4 and offer full GPT-4 capability; however, they employ distinct trained models. CoPilot offers various versions tailored to different tools, each containing unique content for training their models. Although existing studies have compared popular language models like ChatGPT, Gemini, and Copilot, they frequently lack rigorous research design. They are limited to specific medical specialties, such as ophthalmology. There is a noticeable absence of comprehensive studies that evaluate these models across various specialties using robust research methods and statistical analysis. This study aims to fill that gap in literature. With this background, “The study compares the accuracy of ChatGPT, Gemini, and Copilot in answering multiple-choice questions across various specialties by evaluating their responses against those provided by a qualified medical doctor (teacher)”. The questions selected for testing the concordance are taken from sample questions from the United States Medical Licensing Examination (USMLE).

### Research objectives

The research objectives (RO) for this study are three prongs:

- RO1: To assess the accuracy of ChatGPT, Gemini, and Copilot in answering multiple-choice questions (MCQs) compared to a qualified medical teacher.
- RO2: To determine the level of agreement between the responses of the three LLMs and a medical teacher to understand the reliability of AI-generated medical knowledge.
- RO3: To identify which LLM performs best in medical education tasks by analyzing the differences in accuracy and agreement between ChatGPT, Gemini, and Copilot across various medical specialties.

## Methods

### Study design

This study uses a cross-sectional, observational study to measure the consistency of responses among three AI models and a human responder to 40 MCQ questions. The objective of this study includes the evaluation of the concordance among ChatGPT, Gemini, Copilot, and a medical teacher on 40 MCQ responses.

### Participant and instrument

In this study, we evaluated three large language models (LLMs)ChatGPT (GPT-4o), Gemini (1.5 Pro), and Copilot (GPT-4)—alongside a medical educator. We assessed their performance using multiple-choice questions (MCQs) sourced from past United States Medical Licensing Examination (USMLE) materials. These questions were randomly selected to encompass various medical specialties, providing a comprehensive evaluation of each model’s capabilities.

### Data collection

Forty questions were taken from a book containing preparatory material for the United States Medical Licensing Examination. The selected 40 multiple-choice questions used in the study are uploaded to an online repository and available publicly. Each question has several response options (e.g., A, B, C, D, and E). All four respondents (ChatGPT, Gemini, Copilot, and Human) answered the 40 questions independently. The responses from all four respondents were uploaded online and are publicly available.

The sample size for this study comprised 40 multiple-choice questions (MCQs) randomly selected from past United States Medical Licensing Examination (USMLE) preparatory materials. This number was chosen to ensure a diverse representation of medical knowledge across different specialties while maintaining a manageable scope for analysis. A sample of 40 MCQs provides sufficient data to conduct statistical evaluations using Fleiss’ Kappa for overall agreement among responders and Cohen’s Kappa for pairwise comparisons. While a larger sample could improve generalizability, this size allows for meaningful comparison without excessive computational complexity.

### Data analysis

The study first used Fleiss’ Kappa to determine the agreement between four raters. The selection of Fleiss’ Kappa and Cohen’s Kappa in this study is justified based on their appropriateness for assessing agreement among multiple raters and evaluating inter-rater reliability. Fleiss’ Kappa was employed as it extends Cohen’s Kappa to multiple raters, making it suitable for comparing responses from three LLMs (ChatGPT, Gemini, and Copilot) and a medical teacher. This method quantifies the degree of agreement beyond random chance, offering an objective measure of overall concordance. These methods were selected to ensure a statistically sound assessment of AI-generated medical knowledge, highlighting inconsistencies and reinforcing the need for domain-specific fine-tuning of general-purpose LLMs.

Let’s assume the data matrix used for this test is N*k, where N is the number of questions while k is the number of options. In this study, N is 40 while k is 5. For each item, i proportion of $\:{P}_{i}$ is given by:

$$\:{P}_{i}=\frac{1}{n(n-1)}\sum\:_{j=1}^{k}{n}_{ij}({n}_{ij}-1)$$

Where n is the total number of raters. $\:{n}_{ij}$ is the number of raters who assigned category j to item i. $\:{P}_{i}$ measures the degree of agreement for item i. Now $\:{P}_{mean}$ is calculated using the following formula:

$$\:{P}_{mean}=\frac{1}{N}\sum\:_{i=1}^{N}{P}_{i}$$

Once average agreement $\:{P}_{mean}$ is calculated, the next step is to calculate the expected agreement $\:{P}_{e}$. For each category, j, $\:{P}_{j}$ is given by:

$$\:{P}_{j}=\frac{1}{Nn}\sum\:_{i=1}^{N}{n}_{ij}$$

Now, the expected agreement is calculated using the following equation:

$$\:{P}_{e}=\sum\:_{j=1}^{k}{P}_{j}^{2}$$

Finally, Fleiss’ Kappa is calculated using the equation:

$$\:K=\frac{{P}_{mean}-{P}_{e}}{1-{P}_{e}}$$

K > 0.75 indicates excellent agreement among respondents, a value of 0.40 < K ≤ 0.75 represents fair to good agreement, while a value K ≤ 0.40 gives poor agreement between raters [18]. One of the drawbacks of Fleiss’ Kappa is that it doesn’t have any post hoc test, and hence, for comparing the response of AI models and medical teachers, Cohen’s Kappa was used [19]. The following equation gives the Cohen’s Kappa K:

$$\:K=\frac{{P}_{o}-{P}_{e}}{1-{P}_{e}}$$

Where $\:{P}_{o}$ is the observed agreement, while $\:{P}_{e}$ is the expected agreement.

$$\:{P}_{o}=\frac{1}{N}\sum\:_{i=1}^{k}{M}_{ii}$$

Where $\:{M}_{ii}$ is given by the elements where both responders agree. While $\:{P}_{e}$ is given by the following equation:

$$\:{P}_{e}=\sum\:_{i=1}^{k}\frac{{M}_{i}*\:{M}_{i}}{{N}^{2}}\:$$

Where first $\:{M}_{i}$ is given by the total number of items classified in category i by responder one, while second $\:{M}_{i}$ is given by the total number of items classified in category i by responder 2. Again, K > 0.75 indicates excellent agreement among respondents, a value 0.40 < K ≤ 0.75 represents fair to good agreement, while a value K ≤ 0.40 gives poor agreement between raters.

Fleiss’ Kappa assumes independent rates and treats all disagreements equally, which can lead to biased or misleading results, especially in cases with imbalanced category distributions. Interpretation can be challenging, and it does not account for weighted agreements or the varying importance of disagreements [20]. Cohen’s Kappa is limited to pairwise comparisons and assumes equal importance of disagreements, which can result in misleading values, particularly in imbalanced categories (the Kappa Paradox) [21]. It’s sensitive to category prevalence and doesn’t handle ordinal data well, making interpretation difficult without considering context.

## Results

The study evaluated the accuracy of three large language models (LLMs)—ChatGPT (GPT-4o), Gemini (1.5 Pro), and Copilot (GPT-4)—in answering 40 multiple-choice questions (MCQs) from the United States Medical Licensing Examination (USMLE). The responses were compared with those of a qualified medical teacher. The percentage of agreement between each LLM and the medical teacher is summarized in Table 2.

**Table 2:** Agreement between LLMs and the medical teacher

Pair of Responders	Frequency (40)	Percentage
ChatGPT agrees with the Medical Teacher	35	70%
Gemini agrees with the Medical Teacher	25	50%
Copilot agrees with the Medical Teacher	30	60%

Among the three models, ChatGPT demonstrated the highest accuracy at 70%, followed by Copilot at 60%, while Gemini lagged at 50%. This indicates that ChatGPT’s responses were most aligned with expert knowledge, whereas Gemini showed lower concordance.

### Concordance analysis using Fleiss’ kappa

To assess the level of agreement among all four respondents (three LLMs + medical teacher), Fleiss’ Kappa was calculated. The obtained value was − 0.056, indicating poor agreement among the four responders, suggesting that the models provide inconsistent answers, and their reliability varies. Given the low Fleiss’ Kappa value, further pairwise agreement analyses were conducted using Cohen’s Kappa.

### Pairwise agreement using Cohen’s kappa

Pairwise comparisons between each LLM and the medical teacher were assessed using Cohen’s Kappa, providing insights into which model aligns most closely with expert responses. The results are detailed in Table 3.

**Table 3:** Summary of the concordance test between AI models and medical teacher

Pair of Responders	Cohen’s Kappa	Inference
ChatGPT and Gemini	0.470	Fair to good agreement
ChatGPT and Copilot	0.718	Fair to good agreement
ChatGPT and Medical Teacher	0.843	Excellent agreement
Gemini and Copilot	0.526	Fair to good agreement
Gemini and the Medical Teacher	0.531	Fair to good agreement
Copilot and the Medical Teacher	0.688	Fair to good agreement

The result shows that ChatGPT has the highest agreement with the medical teacher (Kappa = 0.843), demonstrating strong reliability in generating accurate responses. Copilot (Kappa = 0.688) performed moderately well but was less consistent than ChatGPT. Finally, Gemini exhibited the lowest agreement (Kappa = 0.531), reinforcing its comparatively weaker performance in answering medical questions.

### Agreement patterns and key observations

**Fig. 2:**
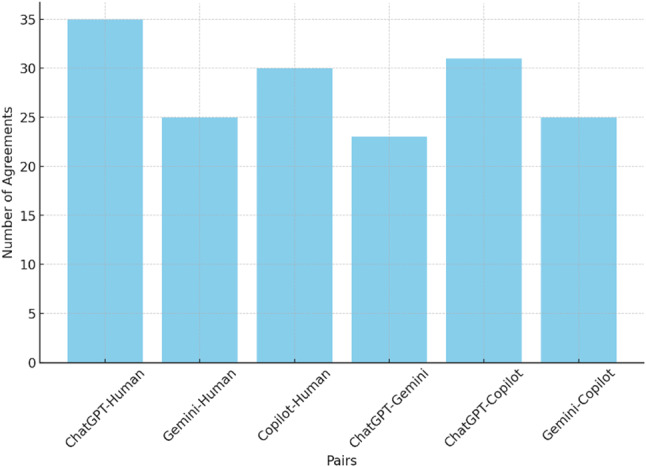
Number of agreements between different Pairs

Figure 2 depicts the agreement frequency between six possible comparison pairs. The figure illustrates that ChatGPT outperforms other models with 35 right answers, while Copilot comes next with 30 right answers. All four responders agree in 21 cases, showing significant discordance among the Gen AI and Medical Teacher.

The results indicate that ChatGPT (GPT-4o) outperformed both Copilot and Gemini, showing the highest concordance with the medical teacher. The low Fleiss’ Kappa value (-0.056) suggests substantial variability in LLM responses, necessitating further refinement for their application in medical education. Cohen’s Kappa analysis confirmed ChatGPT’s reliability, with an agreement score of 0.843, while Gemini’s lower performance suggests it may require domain-specific fine-tuning to improve accuracy.

## Discussions

The study results suggest a lack of concordance between the three Gen AI tools and the medical teacher. Let’s take the medical teacher as an expert and assume her response is correct. ChatGPT outperforms the other two Generative AI models in answering complex questions from the medical entrance examination. This study’s findings align with a recent study that concluded that ChatGPT4 outperformed both Microsoft Copilot and Gemini in accuracy when addressing frequently asked questions in breast imaging, achieving a commendable success rate [22]. Similarly, another study observed that ChatGPT provided 100% correct answers to questions related to methotrexate use, while Gemini scored significantly lower at 73.91%. Lee’s findings further support this superiority in his recent work, where he noted that ChatGPT consistently outperformed Gemini in answering questions related to cardiology [23]. There are some specific areas, such as interpreting clinical biochemical data, where Microsoft Copilot was found useful. A study reports its superiority to ChatGPT-3.5 and Gemini in this domain, suggesting that while ChatGPT excels in general medical inquiries, Copilot may have an edge in specialized biochemical contexts [17]. However, it is to be noted that the performance of Copilot may not be as robust in answering broader medical questions, and it is less likely that Copilot will outperform ChatGPT in answering multiple-choice questions [22]. This finding is parallel with the findings of this study. Google came up with its LLM BARD, which didn’t perform well compared to ChatGPT, forcing it to come up with its improved LLM called Gemini. Gemini is expected to perform better than its competitors but has not lived up to its hefty promise. In a study assessing the readability of patient education materials, Gemini’s responses were less clear than those generated by ChatGPT [24].

Regarding answering the medical question, a study published by Rossettini compares Gemini and ChatGPT [25]. The performance of these models was evaluated based on the Italian entrance test for healthcare sciences. The result of this study again concurs with our study findings that ChatGPT-4 is better at answering a wide range of medical questions than Gemini. Studies report that Copilot is superior to its competitors regarding readability and user engagement [26]. Our experience during the study shows that ChatGPT is comparable in readability and user engagement, if not better than it. Moreover, ChatGPT’s responses are often more detailed and nuanced, which may benefit users seeking in-depth understanding [26]. Large language models (LLMs) in medical contexts exhibit significant limitations and biases that must be critically examined. These models can reproduce existing biases present in training data, leading to inequitable healthcare outcomes, particularly for marginalized groups [27]. Furthermore, the phenomenon of “hallucination,” where LLMs generate inaccurate or misleading information, poses a serious risk in clinical settings, potentially compromising patient care [28]. Additionally, the lack of transparency in LLM decision-making processes complicates the evaluation of their outputs, making it challenging for healthcare professionals to trust these systems [29]. Regular bias audits and clinician oversight are essential to mitigate these risks and ensure that LLMs contribute positively to healthcare [30].

Thus, we can conclude from this study that ChatGPT-4 distinguishes itself among the three AI models—ChatGPT, Gemini, and Microsoft Copilot—due to its exceptional precision and comprehensive knowledge in answering medical questions. Microsoft Copilot demonstrates potential in various domains, especially in the interpretation of biochemical data, whereas Gemini, despite its promise, has not yet attained the same degree of proficiency as ChatGPT. In the context of answering the wider medical questions, ChatGPT looks like the clear winner. The accuracy of 84% compared to the medical teacher suggests that the general purpose LLMs are yet to receive the accuracy desired in medical education and hence need to be used cautiously. RAG (Retrieval-Augmented Generation) tuning is a technique that can enhance the performance of general-purpose large language models (LLMs) by combining retrieval mechanisms with generative models. This approach can significantly improve the accuracy and relevance of the output generated by LLMs, especially in tasks that require detailed, fact-based responses.

## Conclusions

This study performed a comparative analysis of three popular general-purpose LLMs: ChatGPT, Gemini, and Copilot. The study design included randomly selected 40 MCQs from the prestigious USMLE examination. The response of the LLMs was compared with the medical teacher who had post-graduation experience in internal medicine. The study’s findings suggest that none of the LLMs have shown more than 84% accuracy compared to medical teachers. This accuracy shows that general-purpose LLMs must be used cautiously in medical education. The accuracy shown in this study is promising, and these models can be fine-tuned with domain-specific data to improve their accuracy further. One of the possible approaches is the RAG technique. The results show that ChatGPT with Kapps (0.84) is the most accurate, followed by Copilot 0.69. At the same time, Gemini performed worse with the Kappa, with 0.53 accuracy, than the medical teacher. ChatGPT and Copilot have shown good concordance when models are compared with each other, with Kohen’s Kappa being 0.72. While Gemini and ChatGPT have the least agreement, their Cohen’s Kappa value is only 0.47.

### Research implications

This study has three implications for healthcare professionals. Firstly, it advises using general-purpose LLMs with caution in medical education. Secondly, it shows that LLMs such as Gemini perform better in providing domain-specific answers. Finally, this study recommends fine-tuning general-purpose LLMs to domain-specific data to improve their accuracy.

The integration of LLMs in medical education presents ethical and practical challenges. Ethically, the reliance on AI-generated medical knowledge raises concerns about accuracy, misinformation, and patient safety. The inconsistent performance of LLMs suggests that their use should be supplemented with expert validation to prevent potential harm. Practically, while LLMs offer scalable and accessible learning tools, they must be fine-tuned with domain-specific data to enhance reliability. Moreover, educators must train students to critically assess AI-generated content, ensuring informed decision-making rather than blind dependence. Thus, LLMs should be used as an adjunct rather than a replacement for expert medical instruction.

### Limitations of research

The study assumes that the human responder, a qualified medical teacher, serves as the gold standard for evaluating the accuracy of large language models (LLMs). However, interrater variability could arise if different medical experts were used, as their interpretations and knowledge depth may vary. The analysis is confined to 40 MCQs, which, while diverse, may not comprehensively represent all medical specialties. Additionally, some advanced Gen AI tools, such as Perplexity AI, were excluded due to accessibility constraints. The study focuses solely on general-purpose LLMs, without exploring domain-specific fine-tuning. Lastly, it does not analyze the underlying reasons behind the varying performances of the LLMs.

### Future directions

Future research should explore the performance of domain-specific LLMs, such as Med-PaLM, which may offer improved accuracy in medical education. Expanding the study to include a larger and more diverse dataset of MCQs across multiple specialties will provide a more comprehensive evaluation of AI capabilities. Additionally, investigating the impact of fine-tuning general-purpose LLMs with medical datasets can help improve their reliability. Future studies should also assess explainability and reasoning patterns in AI-generated responses. Lastly, comparative studies involving multiple human experts can address interrater variability and ensure a more robust benchmarking framework for AI-assisted medical education.

## Electronic supplementary material

Below is the link to the electronic supplementary material.

Supplementary Material 1

Supplementary Material 2

## Data Availability

All questionnaires and data supporting the reported results are available in the original in the online repository at: https://github.com/vinaytosh/concordance.
