# Supplementary material for: Accuracy of LLMs in medical education: evidence from a concordance test with medical teacher
Source: BMC Med Educ. 2025 Mar 26;25:443. doi: 10.1186/s12909-025-07009-w (PMC11948841; doi:10.1186/s12909-025-07009-w)

USMLE STEP 1 SAMPLE TEST QUESTIONS

BLOCK 1, ITEMS 1-40

1. A 14-year-old boy is brought to the emergency department by his parents because of a 1-month history of intermittent right knee pain that has worsened during the past day. He rates his current pain as a 6 on a 10-point scale and says that it worsens when he walks and lessens when he sits. During the past 2 weeks, he has been walking 1 mile daily in preparation for participation in the school marching band. He has not taken any medications for his pain. He sustained a right tibia and fibula fracture at the age of 8 years after a skateboarding accident, which was treated with internal fixation and casting. He has asthma treated with inhaled budesonide daily and inhaled albuterol as needed. His mother has type 2 diabetes mellitus, and his maternal grandmother has osteoporosis. The patient is 170 cm (5 ft 7 in; 77th percentile) tall and weighs 88 kg (195 lb; >95th percentile); BMI is 31 kg/m^2 (98th percentile). Temperature is 37.0°C (98.6°F), pulse is 95/min, and blood pressure is 130/80 mm Hg. Physical examination shows hyperpigmented, thickened skin at the nape of the neck. There is tenderness to palpation of the anterior aspect of the right hip and limited range of motion on abduction, internal rotation, and flexion of the right hip. The left hip and knees are nontender; range of motion is full in all directions. The remainder of the examination discloses no abnormalities. Which of the following factors in this patient's history most increased his risk for developing this condition?

(A) BMI
(B) Family history
(C) Medication use
(D) Previous fractures
(E) Recent physical activity

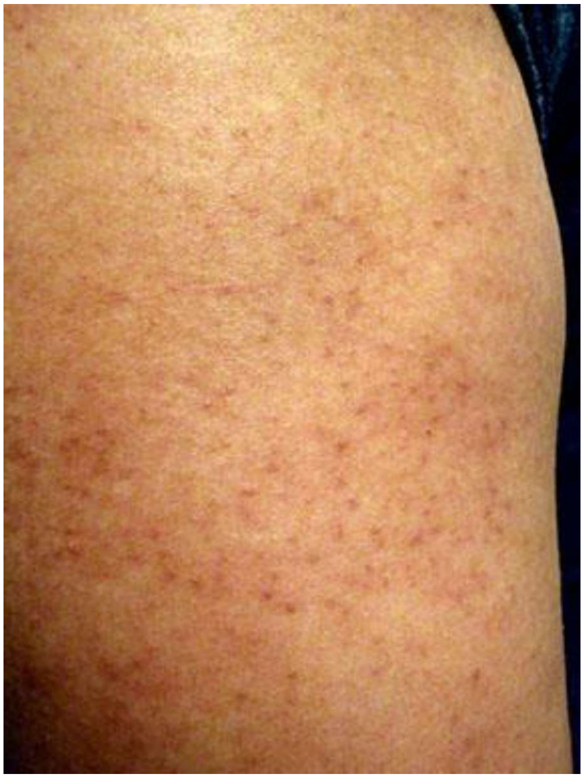

2. A 14-year-old girl is brought to the office by her mother because of a 3-month history of red bumps on her skin. The patient says the bumps are not itchy or painful but that she finds them embarrassing. She has no history of major medical illness and takes no medications. Her vital signs are within normal limits. Physical examination shows the findings in the photograph. Which of the following is the most likely diagnosis?

(A) Eczema
(B) Folliculitis
(C) Hidradenitis
(D) Keratosis pilaris
(E) Urticaria

---

3. A 50-year-old man comes to the office because of a 2-month history of increasing daytime somnolence. He has obstructive sleep apnea for which he has only intermittently used a continuous positive airway pressure device. He is 170 cm (5 ft 7 in) tall and weighs 181 kg (400 lb); BMI is 63 kg/m^2. His temperature is 37°C (98.6°F), pulse is 100/min, respirations are 12/min, and blood pressure is 135/80 mm Hg. Physical examination shows a gray-blue tinge to the lips, earlobes, and nail beds. Cardiac examination shows no other abnormalities. Arterial blood gas analysis on room air shows a pH of 7.31, P_{CO_2} of 70 mm Hg, and P_{O_2} of 50 mm Hg. Which of the following additional findings would be most likely in this patient?

(A) Decreased serum bicarbonate concentration
(B) Increased hemoglobin concentration
(C) Increased total lung capacity
(D) Left ventricular hypertrophy

4. A 32-year-old man comes to the office because of a 1-day history of cough productive of small amounts of blood and a 2-day history of shortness of breath and swelling of his ankles. He also has a 2-week history of progressive fatigue and episodes of dark urine. He has no history of major medical illness and takes no medications. His temperature is 37°C (98.6°F), pulse is 90/min, respirations are 18/min, and blood pressure is 175/110 mm Hg. Pulse oximetry on room air shows an oxygen saturation of 91%. Diffuse inspiratory crackles are heard over all lung bases. There is 2+ pitting edema of both ankles. Results of laboratory studies are shown:

Hemoglobin	8.9 g/dL
Hematocrit	27%
Serum	
Urea nitrogen	55 mg/dL
Creatinine	2.9 mg/dL
Urine RBC	20–40/hpf

Urinalysis also shows some dysmorphic RBCs and rare RBC casts. Examination of a kidney biopsy specimen shows crescentic glomerulonephritis and linear deposition of IgG along the glomerular capillaries. This patient most likely has antibodies directed against which of the following antigens?

(A) Collagen
(B) Double-stranded DNA
(C) Nucleolar protein
(D) Phospholipid
(E) Proteins in neutrophil cytoplasm

---

5. A 5-year-old girl is brought to the emergency department because of a 2-day history of fever, urinary urgency, and burning pain with urination. She has had four similar episodes during the past year. A diagnosis of urinary tract infection is made. Subsequent renal ultrasonography shows one large U-shaped kidney. Which of the following is the most likely embryologic origin of this patient's condition?

(A) Failure of the kidneys to rotate 90 degrees medially
(B) Failure of normal kidney ascent
(C) Failure of one ureteric bud to develop normally
(D) Fusion of the inferior poles of the metanephros during ascent

6. A 78-year-old man comes to the office for a follow-up examination. He was discharged from the hospital 1 week ago after being treated for a nontuberculous mycobacterial infection. He started treatment with ciprofloxacin and rifampin at that time. He also has hypertension and underwent placement of a mechanical aortic valve 6 years ago for aortic stenosis. Other current medications are hydrochlorothiazide, lisinopril, and warfarin. His warfarin dose was doubled 4 days ago. He says that he is trying to follow a healthier diet. He drinks two 12-oz beers daily. Results of laboratory studies done 4 days ago and today are shown

	**4 Days Ago**	**Today**
Prothrombin time	11 sec (INR=1)	11.2 sec (INR=1.1)
Partial thromboplastin time	29 sec	27 sec

Which of the following is the most likely cause of this patient's laboratory findings?

(A) Decreased protein binding
(B) Eradication of gut flora
(C) Increased alcohol intake
(D) Increased vegetable consumption
(E) Induction of cytochrome enzymes

---

7. A 32-year-old man comes to the office because of a 2-week history of fever and throat pain. He is 173 cm (5 ft 8 in) tall and weighs 63 kg (140 lb); BMI is 21 kg/m^2. His pulse is 110/min, respirations are 16/min, and blood pressure is 98/68 mm Hg. Physical examination shows scattered 2- to 4-cm lymph nodes in the neck, axillae, and inguinal regions. There is a bilateral tonsillar exudate but no ulcerations. Results of laboratory studies are shown:

Hemoglobin	9.6 g/dL
Hematocrit	29%
Leukocyte count	1500/mm^3
Platelet count	60,000/mm^3

A heterophile antibody test result is negative. Which of the following is the most likely diagnosis?

(A) Epstein-Barr virus infection
(B) Gonococcal pharyngitis
(C) HIV infection
(D) Lymphogranuloma venereum infection
(E) Streptococcal pharyngitis

---

8. A 50-year-old man comes to the office for a follow-up examination. He has a 2-month history of headache and shortness of breath with exertion. He also has hypertension treated with hydrochlorothiazide for the past 2 years. His blood pressure is 180/105 mm Hg. Ophthalmoscopic examination is most likely to show which of the following in this patient?

(A) Arteriovenous nicking
(B) Melanocytes in the uvea
(C) Optic neuritis
(D) Posterior subcapsular cataracts
(E) Tractional retinal detachment

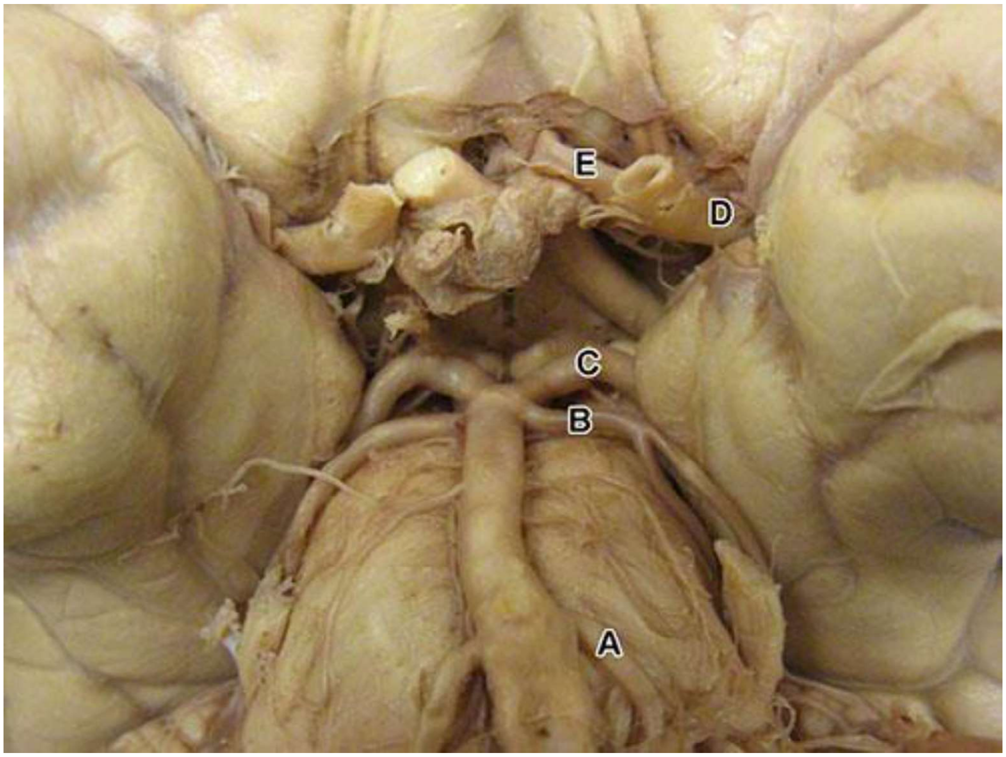

9. A 65-year-old woman with a history of rheumatic mitral valve disease is brought to the emergency department 30 minutes after the sudden onset of right-sided weakness and inability to speak. Neurologic examination shows weakness of the right lower side of the face and difficulty swallowing. Muscle strength is 3/5 on the right side. She can understand what is said to her, but she cannot repeat words or answer questions. An ECG shows atrial fibrillation. The most likely cause of the neurologic findings in this patient is occlusion of which of the following labeled arteries in the photograph of a normal brain?

(A) A
(B) B
(C) C
(D) D
(E) E

---

10. A 51-year-old man with a 10-year history of gastroesophageal reflux and suspected Barrett esophagus comes to the office because his omeprazole dose "doesn't work around the Christmas holidays." He states that he prides himself on having a large appetite and "holding his liquor" during the holidays. He currently takes the maximum dose of omeprazole. Which of the following is the most appropriate initial action by the physician?

(A) Ask the patient how much he is eating and drinking during the holidays
(B) Explain the hazards of untreated reflux in the presence of Barrett esophagus
(C) Order an upper endoscopy
(D) Refer the patient to a gastroenterologist
(E) Switch the omeprazole to pantoprazole

11. A 60-year-old man comes to the office because of weakness, tingling of his hands and feet, irritability, and forgetfulness for 4 months. Physical examination shows pallor, weakness, and spasticity. Deep tendon reflexes are increased. Sensation to vibration is absent in the lower extremities. Laboratory studies show megaloblastic anemia, serum antiparietal cell antibodies, and increased serum concentrations of methylmalonic acid and total homocyst(e)ine. The synthesis of which of the following amino acids is most likely impaired in this patient?

(A) Cysteine
(B) Glutamine
(C) Methionine
(D) Phenylalanine
(E) Tyrosine

12. A 65-year-old woman comes to the office for a follow-up examination 1 year after she underwent operative resection of the right colon and chemotherapy for stage III colon cancer. She reports fatigue. Physical examination shows no abnormalities. A staging CT scan of the chest and abdomen shows five new 2- to 3-cm masses in the liver and both lungs. This patient's cancer most likely spread to the lungs via which of the following structures?

(A) Inferior mesenteric vein
(B) Inferior vena cava
(C) Left colic vein
(D) Middle colic artery
(E) Pulmonary vein
(F) Superior mesenteric artery
(G) Superior vena cava

13. A 26-year-old man comes to the office because of a 1-week history of increased urinary frequency accompanied by excessive thirst. He says he has been urinating hourly. Physical examination shows no abnormalities. Serum chemistry studies are within the reference ranges. Urine osmolality is 50 mOsmol/kg H_2O. After administration of ADH (vasopressin), his urine osmolality is within the reference range. The most likely cause of this patient's symptoms is dysfunction of which of the following structures?

(A) Anterior pituitary gland
(B) Bowman capsule
(C) Glomerulus
(D) Hypophysial portal system
(E) Loop of Henle
(F) Supraoptic nucleus

14. A 52-year-old woman comes to the office because of a 6-month history of intermittent headaches. Sometimes the pain improves when the patient lies down in a quiet room. Her temperature is 37.5°C (99.5°F), pulse is 86/min, respirations are 16/min, and blood pressure is 154/100 mm Hg. The lungs are clear. Cardiac examination shows the point of maximal impulse displaced to the left and occasional skipped beats; there are no murmurs or rubs. There is no S_3. Resting electrocardiography shows left axis deviation with R waves greater than 30 mm in leads V_5 through V_6. Which of the following processes best explains the development of the left ventricular abnormalities in this patient?

(A) Excessive accumulation of glycogen
(B) Fibrosis of intraventricular conduction pathways
(C) Increased synthesis of contractile filaments
(D) Misfolding and aggregation of cytoskeletal proteins
(E) Myocyte hyperplasia as a result of induction of embryonic genes

15. A 53-year-old man comes to the physician because of a dry scaly rash on his body for the past year. He has had a 15-kg (33-lb) weight loss during the past year. He is 178 cm (5 ft 10 in) tall and now weighs 54 kg (120 lb); BMI is 17 kg/m^2. His stools have a large volume and float. Which of the following nutrient deficiencies is most likely?

(A) Magnesium
(B) Vitamin A
(C) Vitamin B_{12} (cobalamin)
(D) Vitamin C
(E) Zinc

16. Serum LDL-cholesterol concentrations are measured in blood samples collected from 25 healthy volunteers. The data follow a normal distribution. The mean and standard deviation for this group are 130 mg/dL and 25 mg/dL, respectively. The standard error of the mean is 5.0. With a 95% confidence level, the true mean for the population from which this sample was drawn falls within which of the following ranges (in mg/dL)?

(A) 105-155
(B) 120-140
(C) 125-135
(D) 128-132
(E) 129-131

17. A 39-year-old man comes to the physician because of a 6-month history of progressive shortness of breath. He has had a cough productive of white sputum for 2 years. He smoked 1 pack of cigarettes daily for 16 years but quit 10 years ago. He is in mild respiratory distress with pursed lips and a barrel chest; he is using the accessory muscles of respiration. Breath sounds are distant and crackles are present in the lower lung fields bilaterally. Pulmonary function tests show a decreased FEV_1:FVC ratio, increased residual volume, and decreased diffusion capacity. An x-ray of the chest shows hyperinflation and hypertranslucency of the lower lobes of both lungs. Which of the following is the most likely diagnosis?

(A) Asthma
(B) Bronchiectasis
(C) Chronic pulmonary fibrosis
(D) Cystic fibrosis
(E) Emphysema

18. Investigators conduct a study that evaluates the effect of finasteride on the incidence of prostate cancer in 500 patients. The investigators recruit an additional 1000 patients for the study. Which of the following effects will this have on the research study?

(A) Greater chance of a Type I error
(B) Greater chance of a Type II error
(C) Less chance of a Type I error
(D) Less chance of a Type II error
(E) Impossible to predict

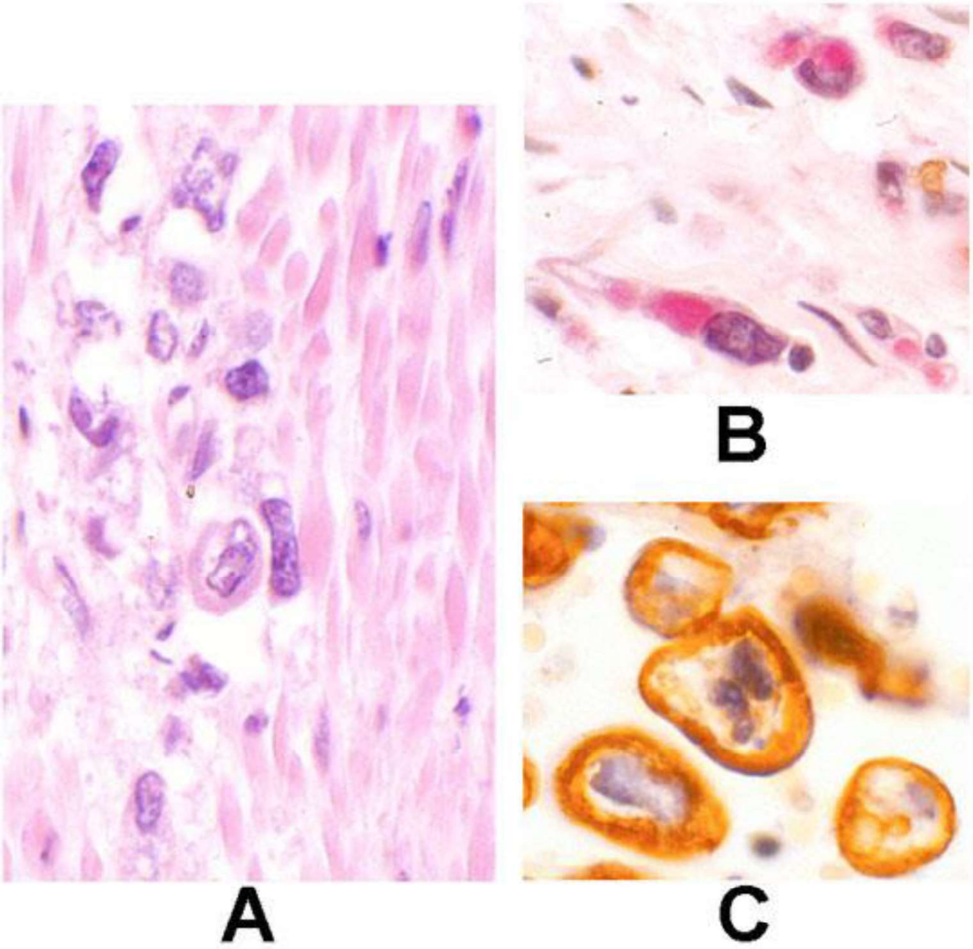

19. An 82-year-old woman has a 7-month history of increasing indigestion, upper abdominal bloating, and early satiety. Photomicrographs of her gastric wall are shown: A is a hematoxylin and eosin stain; B is a mucin stain; C is an immunostain for cytokeratin. Which of the following is most likely to have decreased expression in these cells?

(A) Cathepsin D
(B) Epithelial cadherins
(C) Heparin-binding fibroblast growth factors
(D) Integrins
(E) Type IV collagenase
(F) Vascular endothelial growth factor

---

20. A 54-year-old woman comes to the physician because she would like to lose weight. She has been on numerous diets in the past with limited success. Both her parents have type 2 diabetes mellitus. She is 160 cm (5 ft 3 in) tall and weighs 69 kg (152 lb); BMI is 27 kg/m^2. Her blood pressure is 140/90 mm Hg. Fasting serum glucose concentration is 102 mg/dL. Compared with a woman of the same age whose weight is normal, which of the following serum abnormalities is most likely in this patient?

(A) Decreased cholesterol excretion
(B) Decreased estrone concentration
(C) Decreased leptin concentration
(D) Increased fasting insulin concentration
(E) Increased growth hormone concentration
(F) Increased thyroid-stimulating hormone concentration

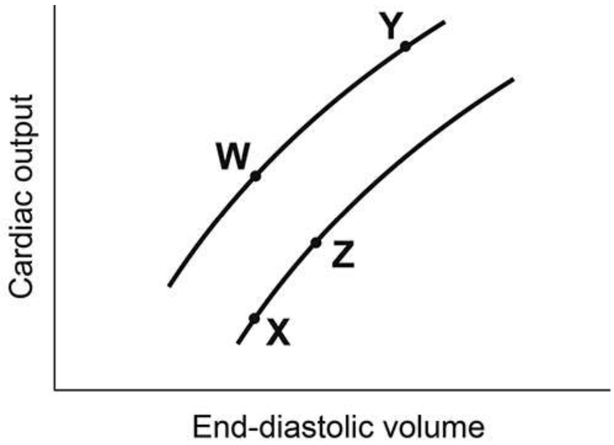

21. A 65-year-old woman is brought to the emergency department because of a 10-minute history of chest tightness and severe pain of her left arm. Physical examination shows jugular venous distention. Crackles are heard over the lung fields. An ECG shows ST-segment elevation greater than 1 mm in leads V_4 through V_6 and new Q waves. Serum studies show an increased troponin I concentration. Which of the following labeled points in the graph best represents the changes in cardiac function that occurred during the first 10 seconds after the onset of pain in this patient?

(A) W → X
(B) W → Y
(C) W → Z
(D) X → W
(E) X → Y
(F) X → Z
(G) Z → W
(H) Z → X
(I) Z → Y

---

22. A previously healthy 33-year-old woman is brought to the emergency department by the Secret Service for stalking the president of the USA for 2 months. She claims to be married to the president's twin brother and states that the president just had his twin kidnapped to avoid competition. She speaks rapidly and is difficult to interrupt. Her associations are often loose. She says, "I haven't slept for days, but I won't even try to sleep until my husband is rescued. God has been instructing me to take over the White House. I can't wait to be reunited with my husband. I hear his voice telling me what to do." When asked about drug use, she says she uses only natural substances. She refuses to permit blood or urine tests, saying, "I don't have time to wait for the results." Which of the following is the most likely diagnosis?

(A) Bipolar disorder, manic, with psychotic features
(B) Brief psychotic disorder
(C) Delusional disorder
(D) Psychotic disorder due to general medical condition
(E) Schizophrenia

23. In informing a couple that their newborn has Down syndrome, there is a specific, relatively limited amount of information that the consulting physician should give immediately. The rest can be discussed at a later time. Which of the following best explains the purpose of using this approach to disclosure?

(A) Allowing the couple's primary care physician to discuss most of the information with them
(B) Allowing the parents time to tell other family members
(C) Delaying parental distress until the information is completely disclosed
(D) Disclosing the most important information so that it can be understood as fully as possible
(E) Influencing the parents' course of action about what is medically most appropriate

24. A 62-year-old man comes to the physician because of a 6-month history of urinary hesitancy and dribbling after urination. He has to urinate two to three times nightly. Physical examination shows a diffusely enlarged, firm, and nontender prostate. Which of the following is most likely to have contributed to the development of this patient's condition?

(A) Activation of the α_1-adrenergic receptor
(B) Conversion of testosterone to dihydrotestosterone
(C) Conversion of testosterone to estradiol
(D) Inhibition of the α_1-adrenergic receptor
(E) Production of prostate-specific antigen

25. A 19-year-old man who is in the US Army is brought to the emergency department 45 minutes after he sustained a knife wound to the right side of his chest during an altercation. He has no history of major medical illness and takes no medications. His temperature is 36.9°C (98.4°F), pulse is 110/min, respirations are 24/min, and blood pressure is 114/76 mm Hg. Pulse oximetry on room air shows an oxygen saturation of 94%. On physical examination, the trachea appears to be shifted to the left. Pulmonary examination of the right chest is most likely to show which of the following findings?

	**Fremitus**	**Percussion**	**Breath Sounds**
(A)	Decreased	dull	decreased
(B)	Decreased	hyperresonant	decreased
(C)	Decreased	hyperresonant	dull
(D)	Increased	dull	bronchial
(E)	Increased	dull	decreased

---

26. A 34-year-old man comes to the office because of a 1-month history of diarrhea. He has a history of pheochromocytoma treated 2 years ago. His mother is being treated for a tumor of her parathyroid gland. He has no other history of major medical illness and takes no medications. His temperature is 37.0°C (98.6°F), pulse is 84/min, respirations are 10/min, and blood pressure is 120/75 mm Hg. Pulse oximetry on room air shows an oxygen saturation of 97%. Vital signs are within normal limits. Physical examination shows a 3-cm, palpable mass on the right side of the neck. A biopsy specimen of the mass shows a neuroendocrine neoplasm of parafollicular cell origin. The most likely cause of the findings in this patient is a mutation in which of the following types of genes?

(A) Cell cycle regulation gene
(B) DNA mismatch repair gene
(C) Metastasis suppressor gene
(D) Proto-oncogene
(E) Tumor suppressor gene

27. A 56-year-old man is brought to the emergency department by his wife 30 minutes after he had severe upper back pain and hoarseness before becoming comatose. He has hypertension treated with hydrochlorothiazide. His temperature is 37°C (98.6°F), pulse is 100/min, and blood pressure is 160/80 mm Hg in the right arm and 100/60 mm Hg in the left arm. Ophthalmologic examination shows ptosis and miosis of the left eye. There is anhidrosis of the left side of the forehead, right hemiplegia, and a decreased left radial pulse. A chest x-ray shows a widened mediastinum. Which of the following conditions is the most likely cause of these findings?

(A) Dissection of the aorta distal to the left subclavian artery
(B) Dissection of the aorta extending into the left carotid artery
(C) Dissection of the aorta extending into the left carotid artery and distal aortic arch
(D) Dissection of the proximal aorta extending into the right subclavian artery
(E) Superior sulcus tumor
(F) Thrombus of the left carotid artery

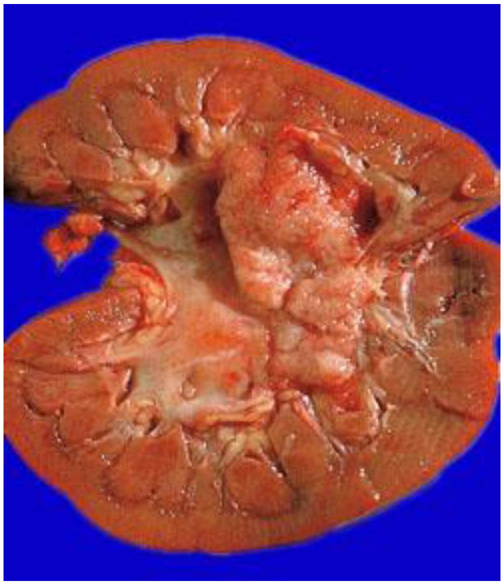

28. A 68-year-old man with alcohol use disorder comes to the office because of a 3-month history of intermittent blood in his urine; he has had no pain. He is a retired laboratory technician from a company that produces naphthylamine. He has smoked 1½ packs of cigarettes daily for 45 years. A CT scan of the abdomen shows a mass in the pelvis of the left kidney. A photograph of the surgically resected kidney is shown. The neoplastic process in this kidney is most likely to be which of the following?

(A) Angiomyolipoma
(B) Metastatic melanoma
(C) Nephroblastoma
(D) Oncocytoma
(E) Urothelial carcinoma

---

29. A 27-year-old woman comes to the emergency department because of a 1-hour history of severe shortness of breath. She has just returned from a cross-country flight. She has a history of borderline hypertension. Her temperature is 36.9°C (98.5°F), pulse is 113/min, respirations are 28/min, and blood pressure is 138/85 mm Hg. Physical examination shows that the right calf has an increased circumference compared with the left calf, and there is tenderness behind the right knee. Which of the following is the most likely underlying cause of this patient's condition?

(A) Antithrombin III deficiency
(B) Factor V Leiden mutation
(C) Glanzmann thrombasthenia
(D) Protein C deficiency
(E) von Willebrand disease

30. A 20-year-old woman is brought to the urgent care center because of a 2-month history of progressive weakness of her arms. She also has a 1-week history of moderate back pain and headache. Her only medication is ibuprofen as needed for pain. Muscle strength is 3/5 in the upper extremities. Sensation to pinprick is decreased over the upper extremities. MRI of the spine shows a central syrinx in the cervical spinal cord. It is most appropriate to obtain specific additional history regarding which of the following in this patient?

(A) Diet
(B) Family illness
(C) Recent travel
(D) Trauma
(E) Unintended weight loss

31. A 3-year-old boy is brought to the office because of a 2-day history of bulging of his left eye. He says his eye hurts. He has no history of major medical illness or recent trauma to the area, and he receives no medications. Vital signs are within normal limits. Physical examination shows exophthalmos of the left eye. MRI of the brain shows a 2-cm mass involving the ocular muscles of the left eye. A biopsy specimen of the mass shows malignant cells, some of which have striations. Which of the following is the most likely diagnosis?

(A) Neuroblastoma
(B) Pheochromocytoma
(C) Retinoblastoma
(D) Rhabdomyosarcoma
(E) Thyroid cancer

32. An 18-year-old woman with sickle cell disease is brought to the emergency department by her parents because of a 2-hour history of severe abdominal pain and nausea. Her parents say that she had a cheeseburger, milk shake, and chocolate bar for lunch. Her temperature is 37°C (98.6°F). Physical examination shows tenderness over the right upper quadrant of the abdomen, radiating to the right shoulder. Ultrasonography of the right upper quadrant of the abdomen shows gallstones. Which of the following is the most likely underlying cause of this patient's current condition?

(A) Decreased hepatic secretion of lecithin
(B) Decreased reabsorption of bile salts
(C) High ratio of cholesterol to bile acids in bile
(D) Infestation with parasites secreting β-glucuronidase
(E) Overload of unconjugated bilirubin

33. In a sample of 100 individuals, the mean leukocyte count is $7500/mm^3$, with a standard deviation of $1000/mm^3$. If the leukocyte counts in this population follow a normal (gaussian) distribution, approximately 50% of individuals will have which of the following total leukocyte counts?

(A) $5500–9500/mm^3$
(B) $<6500/mm^3$ or $>8500/mm^3$
(C) $6500–8500/mm^3$
(D) $<7500/mm^3$
(E) $>9500/mm^3$

34. A previously healthy 52-year-old woman comes to the physician because of a 2-month history of fatigue, constipation, and frequent urination. Her temperature is 37.1°C (98.8°F), pulse is 80/min, respirations are 14/min, and blood pressure is 140/90 mm Hg. Diffuse crackles are heard bilaterally. Her serum calcium concentration is 11.1 mg/dL, and serum parathyroid hormone concentration is decreased. A chest x-ray shows bilateral hilar lymphadenopathy and interstitial infiltrates. Which of the following is the most likely cause of this patient's hypercalcemia?

(A) Calcitriol production by activated macrophages
(B) Local resorption of bone by metastases
(C) Parathyroid hormone-related peptide secretion
(D) Secretion of parathyroid hormone
(E) Secretion of thyroid-stimulating hormone

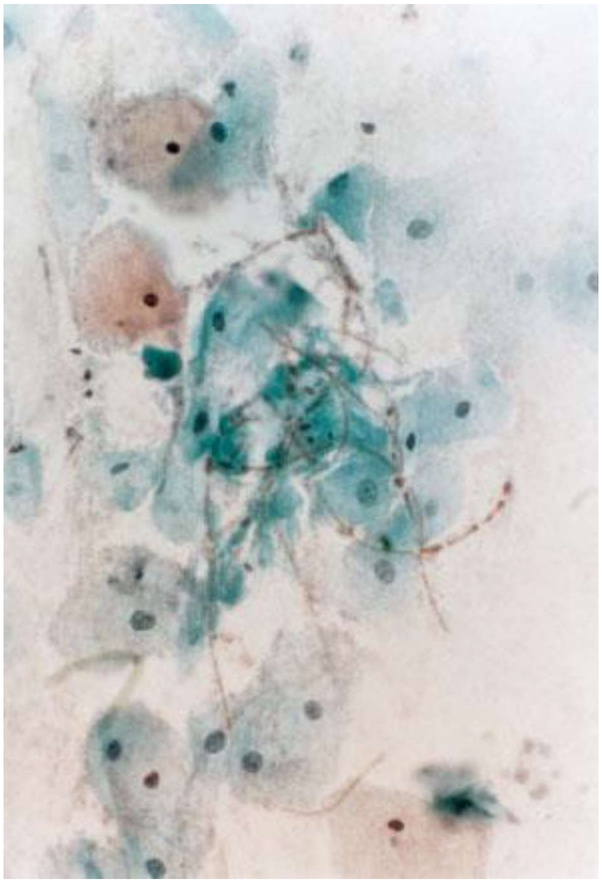

35. A 55-year-old woman comes to the clinic because of a 2-month history of increasingly severe vaginal pain and itching during sexual intercourse. She avoids intercourse with her husband because of the symptoms. She has been in a monogamous relationship with her husband for the past 25 years. She has type 2 diabetes mellitus. Her vital signs are within normal limits. Pelvic examination shows edematous and erythematous vaginal mucosa with white discharge. A photomicrograph of a vaginal smear is shown. Which of the following is the most likely causal infectious agent?

(A) *Candida albicans*
(B) *Chlamydia trachomatis*
(C) Herpes simplex virus
(D) Human papillomavirus
(E) *Trichomonas vaginalis*

---

36. A 24-year-old woman is brought to the physician 1 month after she was involved in a motor vehicle collision that left her weak and unable to walk. Physical examination shows weakness of both hands and atrophy of the intrinsic hand muscles bilaterally. There is weakness and increased muscle tone of the lower extremities on passive range of motion. Deep tendon reflexes are normal at the biceps and triceps bilaterally and are increased at the knees and ankles. Babinski sign is present bilaterally. Sensation to pinprick is absent at and below the level of the clavicles. The lesion in this patient is most likely located at which of the following spinal cord levels?

(A) C5
(B) C7
(C) T1
(D) T3
(E) T5

37. A 45-year-old man is brought to the emergency department 30 minutes after the sudden onset of crushing chest pain. His father, maternal aunt, and paternal uncle all died of myocardial infarctions under the age of 50 years. Physical examination shows tendinous xanthomas on the hands and thickened Achilles tendons. Serum lipid studies show a total cholesterol concentration of 410 mg/dL, HDL-cholesterol concentration of 30 mg/dL, and triglyceride concentration of 140 mg/dL. The diagnosis of myocardial infarction is made. This patient most likely has a deficiency of which of the following?

(A) Apo B48
(B) Apo C
(C) HMG-CoA reductase activity
(D) LDL receptor
(E) Lipoprotein lipase activity

38. A previously healthy 19-year-old man is brought to the emergency department 30 minutes after he collapsed while playing softball. He had severe, sharp, upper back pain prior to the game. He is 196 cm (6 ft 5 in) tall. His temperature is 37°C (98.6°F), pulse is 130/min, respirations are 24/min, and blood pressure is 80/50 mm Hg. Physical examination shows pallor and no jugular venous distention. Breath sounds are clear. The carotid pulses are weak. A grade 4/6, late diastolic murmur is heard at the lower left sternal border. Which of the following is the most likely cause of this patient's cardiac findings?

(A) Atrial septal defect
(B) Mitral stenosis
(C) Papillary muscle rupture
(D) Perforated tricuspid valve
(E) Stretched aortic anulus

39. A 27-year-old woman is brought to the emergency department because of a 2-week history of double vision. Neurologic examination shows that the left eye does not adduct past the midline on horizontal gaze when the patient looks to the right. Leftward horizontal gaze is normal. This patient's ocular movement deficit is most likely caused by damage to which of the following structures?

(A) Left abducens nerve
(B) Left medial longitudinal fasciculus
(C) Left nucleus of the abducens nerve
(D) Right abducens nerve
(E) Right medial longitudinal fasciculus
(F) Right nucleus of the abducens nerve

40. A 45-year-old woman is recovering from acute respiratory distress syndrome secondary to gallstone pancreatitis. A drawing of the alveolar wall is shown. Which of the following labeled cells will most likely proliferate and reestablish the injured epithelial layer in this patient?

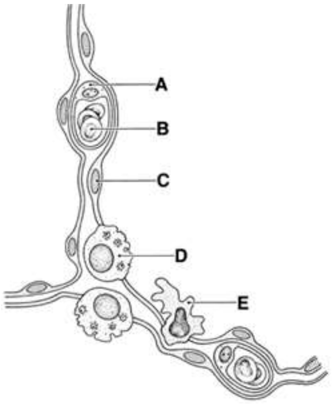

Supplement: Supplementary file 2 — Supplementary Material 2 [file 12909_2025_7009_MOESM2_ESM.pdf]
